# Correlation of low serum vitamin-D with uterine leiomyoma: a systematic review and meta-analysis

**DOI:** 10.1186/s12958-020-00644-6

**Published:** 2020-08-14

**Authors:** Ramin Mohammadi, Reza Tabrizi, Kamran Hessami, Hoda Ashari, Peyman Nowrouzi-Sohrabi, Mahnaz Hosseini-Bensenjan, Nasrin Asadi

**Affiliations:** 1 Student Research Committee, Shiraz University of Medical Sciences, Shiraz, Iran; 2 Health Policy Research Center, Institute of Health, Shiraz University of Medical Sciences, Shiraz, Iran; 3 Maternal-Fetal Medicine Research Center, Shiraz University of Medical Sciences, Shiraz, Iran; 4 Department of Biochemistry, School of Medicine, Shiraz University of Medical Sciences, Shiraz, Iran

**Keywords:** Vitamin D, Uterine fibroid, Uterine leiomyoma, Meta-analysis, Systematic review

## Abstract

**Background:**

There is a growing body of evidence on low serum vitamin-D levels and the risk of uterine leiomyomas (UL). Therefore, this systematic review and meta-analysis was conducted to investigate the association between serum vitamin D levels and UL occurrence.

**Methods:**

Searches were systematically conducted of the electronic databases PubMed, Scopus, EMBASE, Web of Science (ISI), Cochrane library, Ovid, and Google Scholar to identify relevant studies from inception until February 6, 2020. Heterogeneity across the included studies was examined using Cochran’s Q and I-square (I^2^). Data was pooled using random effects modeling and expressed as standardized mean differences (SMDs).

**Results:**

Nine eligible studies with a total of 1730 participants (835 patients with UL and 895 controls) were included in the current meta-analysis. Pooled results with random effects modeling indicated that serum vitamin D levels were significantly lower in patients with UL than in the control group (*n* = 9, SMD = − 0.67; 95% CI, − 0.98, − 0.35, *p* < 0.001; I^2^ = 89.3%, *p* < 0.001). Based on the findings of subgroup analyses, it was found that the SMD values across the included studies from Asia (*n* = 4, SMD = − 1.20; 95% CI, − 1.45, − 0.96, *p* < 0.001; I^2^ = 30.6%, *p* = 0.229) were lower than those from Europe (*n* = 3, SMD = − 0.34; 95% CI, − 0.49, − 0.18, *p* < 0.001; I^2^ = 0.0%, *p* = 0.602) and Africa (*n* = 2, SMD = − 0.13; 95% CI, − 0.29, 0.04, *p* = 0.128; I^2^ = 0.0%, *p* = 0.417), although the difference was not significant in Africa. Publication year was also found to be a potential contributor’s variable in the pooled SMD using the meta-regression method (t = − 3.00, *p* = 0.02).

**Conclusions:**

To the best of our knowledge, the current meta-analysis showed for the first time that serum vitamin D levels were significantly lower in women with UL in selected populations.

## Introduction

The term uterine leiomyoma (UL), also called uterine fibroids, represents the most common benign gynecological tumor affecting the female reproductive system and primarily originates from uterine smooth muscles [1]. The prevalence of UL has been estimated to range from approximately 5 to 69%, depending on the population [1, 2]. The prevalence of UL increases with age throughout the reproductive years; then it declines after menopause [3]. UL causes a substantial economic burden on the healthcare system. Accordingly, the annual economic burden of UL has been assessed to be $34.4 billion US dollars in the United States, which is estimated to be higher than that of breast, colon, or ovarian cancers [4]. Furthermore, a previous review of the literature showed that UL is associated with substantial direct and indirect costs, ranging from approximately $2000 to $16,000 US dollars per patient, for healthcare payers and society [5].

Despite identifying risk factors such as age, black race, premenopausal state, family history, hormonal factors, obesity, and nulliparity that play a role in the development of UL, the exact pathogenesis of this condition remains poorly understood [1, 2, 6]. Interestingly, recent evidence has suggested that vitamin D deficiency, a growing health concern with various consequences such as increased risk of female reproductive tumors [7], has a potential role in the pathogenesis and growth of UL. The activity of vitamin D against UL cells has been supported by various in vitro and in vivo studies [8–10]. The various effects of vitamin D are mainly mediated through activating vitamin D receptors (VDRs). In this regard, VDRs are expressed in myometrial, endometrial, and UL monoclonal tumor cells [11]. Previous reports have highlighted the fact that transforming growth factor beta (TGF-β), involved in the development and progression of UL, can be diminished by the active metabolite of vitamin D [9].

The in vitro study by Blauer et al. was one of the first reports to show that both myometrial and UL cell growth were effectively inhibited by active metabolite of vitamin D and concluded that it may play a role in the growth of UL [8]. Following this, Corachan et al. demonstrated that vitamin D acts as an antiproliferative, antifibrotic, and proapoptotic agent in a xenograft animal model and has the potential to be used as a treatment option for safely reducing UL size [12]. In a case-control study performed on 90 women, the mean concentration of 25-(OH) D3 was significantly lower in women with UL compared with the controls (*p* < 0.001). In the latter study, the UL size increased proportionately with the decrease in 25-(OH) D3 levels (*p* = 0.014) [13]. In another study on 124 women (*n* = 56 UL and *n* = 68 healthy control), the mean level of 25-(OH) D3 was also significantly lower among those with UL; however, vitamin D level was not correlated with the size or volume of UL [14]. Conversely, Mitro et al. indicated that there was no significant relationship between vitamin D levels and the odds of developing UL [15]. However, the biggest limitation of the latter study was that patients’ self-reported UL was used as an outcome measure, and ultrasound documentation was not applied.

For this reason, the current study aimed to assess the potential correlation between serum vitamin D level and the risk of UL in this meta-analysis and systematic review.

## Materials and methods

The results of this study are reported according to the guidelines outlined in the preferred reporting items for systematic reviews and meta-analyses (PRISMA) [16].

### Search strategy

Searches were conducted systematically of the electronic databases including PubMed, Scopus, EMBASE, Web of Science (ISI), Cochrane library, Ovid, Google scholar to identify relevant studies that have been investigated the association between serum vitamin D levels and UL from inception until 6th February 2020.

Search strategy were performed using the following text words and MeSH terms: [“Leiomyoma” OR “Uterine Fibroid” OR “Leiomyomas” OR “Fibromyoma” OR “Fibromyomas” OR “Fibroid” OR “Fibroids” OR “Fibroma” OR “Fibromas” OR “Leiomyomatosis” OR “Leiomyomatoses” OR “myoma”] AND [“Vitamin D” OR “Cholecalciferol” OR “Calciol” OR “Vitamin D 3” OR “Vitamin D3” OR “Cholecalciferols” OR “Hydroxycholecalciferols” OR “Hydroxyvitamins D” OR “Hydroxycholecalciferol” OR “Calcifediol” OR “25-Hydroxyvitamin D 3” OR “25 Hydroxyvitamin D 3” OR “25-Hydroxycholecalciferol” OR “Calcidiol” OR “Hydroxycholecalciferol” OR “Dedrogyl” OR “Hidroferol” OR “Calderol” OR “Dihydroxycholecalciferols” OR “Dihydroxyvitamins D” OR “24,25-Dihydroxyvitamin D 3” OR “Dihydroxyvitamin” OR “24,25-Dihydroxyvitamin” OR “24,25-Dihydroxycholecalciferol” OR “Dihydroxyvitamin” OR “Calcitriol” OR “1 alpha,25-Dihydroxyvitamin” OR “1,25-Dihydroxyvitamin” OR “1,25-Dihydroxyvitamin” OR “1 alpha,25-Dihydroxycholecalciferol” OR “1,25-Dihydroxycholecalciferol” OR “Dihydroxycholecalciferol” OR “Bocatriol” OR “Calcijex” OR “Decostriol” OR “MC1288” OR “MC-1288” OR “MC 1288” OR “Osteotriol” OR “Renatriol” OR “Rocaltrol” OR “Silkis” OR “Sitriol” OR “Soltriol” OR “Tirocal”]. To increase the sensitivity of our search results, we checked manually the reference lists of relevant pervious reviews and eligible studies.

### Study selection

Studies were included that met the following inclusion criteria: study was an observational human article (with cohort, case-control, or cross-sectional design) in English language; investigated the association between serum vitamin D levels and UL; and reported sufficient data to calculate mean difference with 95% confidence intervals (95% CI) for investigating serum vitamin D levels in patients with UL group compared with control group without UL. Studies such as case report, case series, animal study, letter to editor, review study, abstracts without full text, and studies that did not control group were excluded.

### Data extraction

Data extraction was performed by two independent authors (M-H.B and P-N.S) from the included articles using standard sheet form of Microsoft Excel. Any discrepancies were resolved through censuses or discussion with a three author (RT or NA).

The following data were extracted: the first author’s name, year of publication, study design, study setting, participant characteristics (case and control groups), method of vitamin D assessment, number of patients in UL group and control group, and mean (SD) vitamin D levels in case and control group.

### Quality assessment

The Newcastle-Ottawa Scale were used to assess the quality of included studies. This scale assesses using three aspects: “participant selection, comparability of study groups and assessment of outcome or exposure”. Study with a NOS scored ≥7 was considered as a high quality [17].

### Statistical analysis

The overall pooled effect size was indicated by standardized mean differences (SMDs) using the Hedges method. A random effects model was used to combine SMDs with the DerSimonian and Laird method. The Cochran (Q) statistic and I2 test were applied to assess heterogeneity among the included studies. A Q test of *p* < 0.1 and I2 ≥ 50% was considered to be significant heterogeneity across the included studies [18]. Sensitivity analyses were conducted to assess the influence of one-by-one study on the overall pooled SMDs after excluding each study using the leave-one-out method. Finally, subgroup analyses [including study area (Europe vs. Africa vs. Asia) and quality score (high vs. low)] and the meta-regression method [based on publication year and sample size] were applied to detect the source of heterogeneity according to suspected potential variables. Begg’s rank correlation and Egger’s regression tests were used to verify the evidence of publication bias [19, 20]. Statistical analyses were conducted using the STATA version 12.0 (Stata Corp., College Station, TX) software package.

## Results

### Characteristics of included studies

**Fig. 1:**
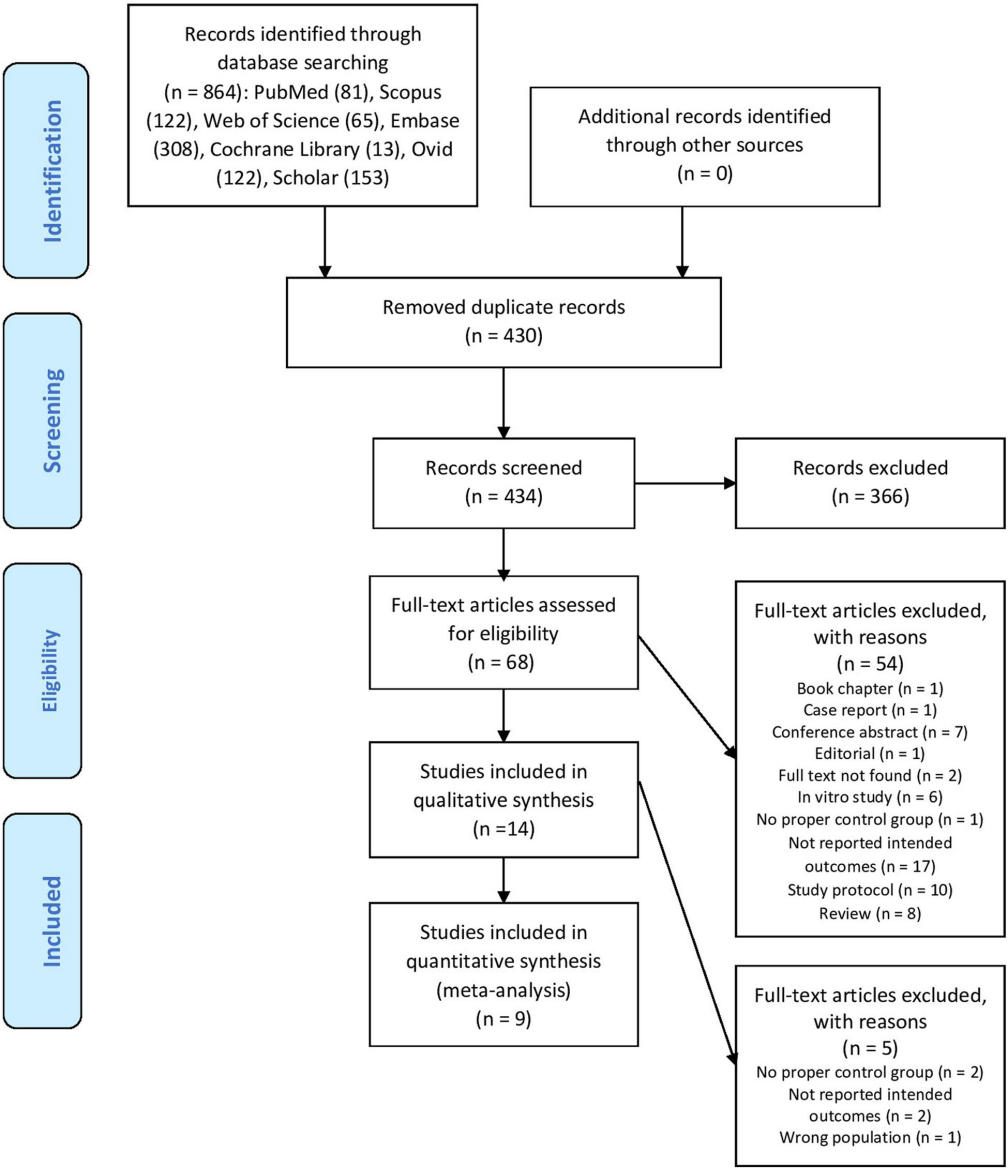
Flowchart of study identification and selection process

In the initial electronic database searches, 864 reports were identified as being related to serum vitamin D levels and UL. After removing 430 duplicates, 434 reports were screened based on titles/abstracts. Of these, 68 full-text articles were retrieved to be assessed for eligibility based on the study’s inclusion criteria. Finally, 9 articles (4 case-control [21–24] and 4 cross-sectional [13, 14, 25, 26] and 1 retrospective cohort study [27] were selected for the current meta-analysis. Figure 1 illustrates the steps of the study identification and selection process in this review.

The nine included articles comprised 1730 participants (ranging from 64 to 432), numbering 835 and 895 in case and control groups, respectively. The included studies were published between 2013 [21, 25] and 2019 [13]; three studies were conducted in India [13, 23, 26], and one each in Italy [21], Egypt [25], Poland [27], Congo [22], Iraq [24], and Turkey [14]. The characteristics of the nine included articles with more details are shown in Table 1.

**Table 1:** Characteristics of included studies

Authors	Publication year	Country	Sample size (Case/Control)	Study design	Method of vitamin d assay	Age group (Control vs. case)	Quality assessment (score)	Reference
Paffoni et al	2013	Italy	128/256	Case-control study	chemiluminescence	37.8 ± 3.1, 37.8 ± 3.1	8	[21]
Sabry et al	2013	Egypt	104/50	Cross-sectional study	radioimmunoassay	36.8 ± 3.4, 37.1 ± 2.9	8	[25]
Ciebiera et al	2016	Poland	105/83	Cohort study	ELISA	42.4 ± 11.2, 42.9 ± 8.1	7	[27]
Ingala et al	2016	Congo	216/216	Case-control study	immunoradiometric assay (= radioimmunoassay)	37.20 ± 12.36, 37.78 ± 8.22	8	[22]
Dawood et al	2017	Iraq	34/30	Case-control study	ELISA	40–50	8	[24]
Oskovi Kaplan et al	2017	Turkey	56/68	Cross-sectional study	electrochemiluminescence immunoassay (ECLIA)	39.84 ± 3.99, 38.25 ± 4.88	8	[14]
Ajmani et al	2018	India	75/75	Case-control study^a^	chemiluminescence assay	38.6 ± 7.78, 36.79 ± 8.97	5	[23]
Singh et al	2018	India	72/72	Cross-sectional study	chemiluminescence immunoassay	42.18 ± 5.37, 41.79 ± 4.91	4	[26]
Srivastava et al	2019	India	45/45	Cross-sectional study	Chemiluminescence	38.47 ± 6.23, 38.27 ± 5.93	5	[13]

^a^study design perceived from methods

### Main outcomes

**Fig. 2:**
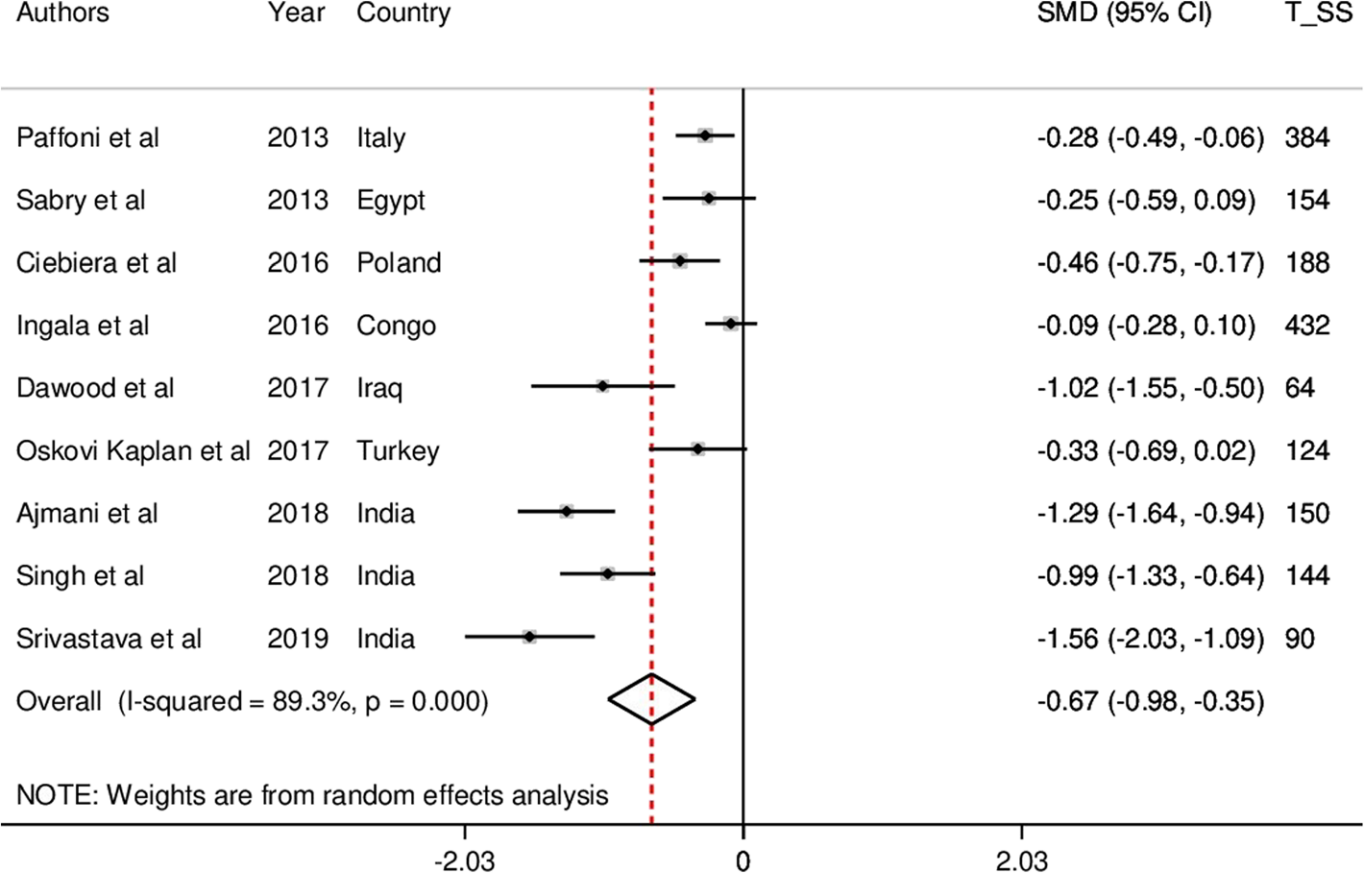
The forest plot for serum vitamin D levels in uterine leiomyoma patients and controls

The forest plots for serum vitamin D levels in UL patients and controls are presented in Fig. 2. The pooled results with random effects modeling showed that serum vitamin D levels were significantly lower in patients with UL (SMD = − 0.67; 95% CI, − 0.98, − 0.35, *p* < 0.001; I^2^ = 89.3%, *p* < 0.001) compared with the control group.

Because of the existence of significant heterogeneity (I^2^ = 89.3%, *p* < 0.001), subgroup analyses were conducted based on potential suspected variables, including study area (Europe vs. Africa vs. Asia) and quality score (high vs. low). It was found that heterogeneity was decreased based on study area. Moreover, the SMD values across the included studies from Asia (*n* = 4, SMD = − 1.20; 95% CI, − 1.45, − 0.96, *p* < 0.001; I^2^ = 30.6%, *p* = 0.229) were higher than those from Europe (*n* = 3, SMD = − 0.34; 95% CI, − 0.49, − 0.18, *p* < 0.001; I^2^ = 0.0%, *p* = 0.602) and Africa (*n* = 2, SMD = − 0.13; 95% CI, − 0.29, 0.04, *p* = 0.128; I^2^ = 0.0%, *p* = 0.417). Although the difference was not significant in Africa, classification of pooled SMD according to quality score did not affect heterogeneity across the included studies.

**Fig. 3:**
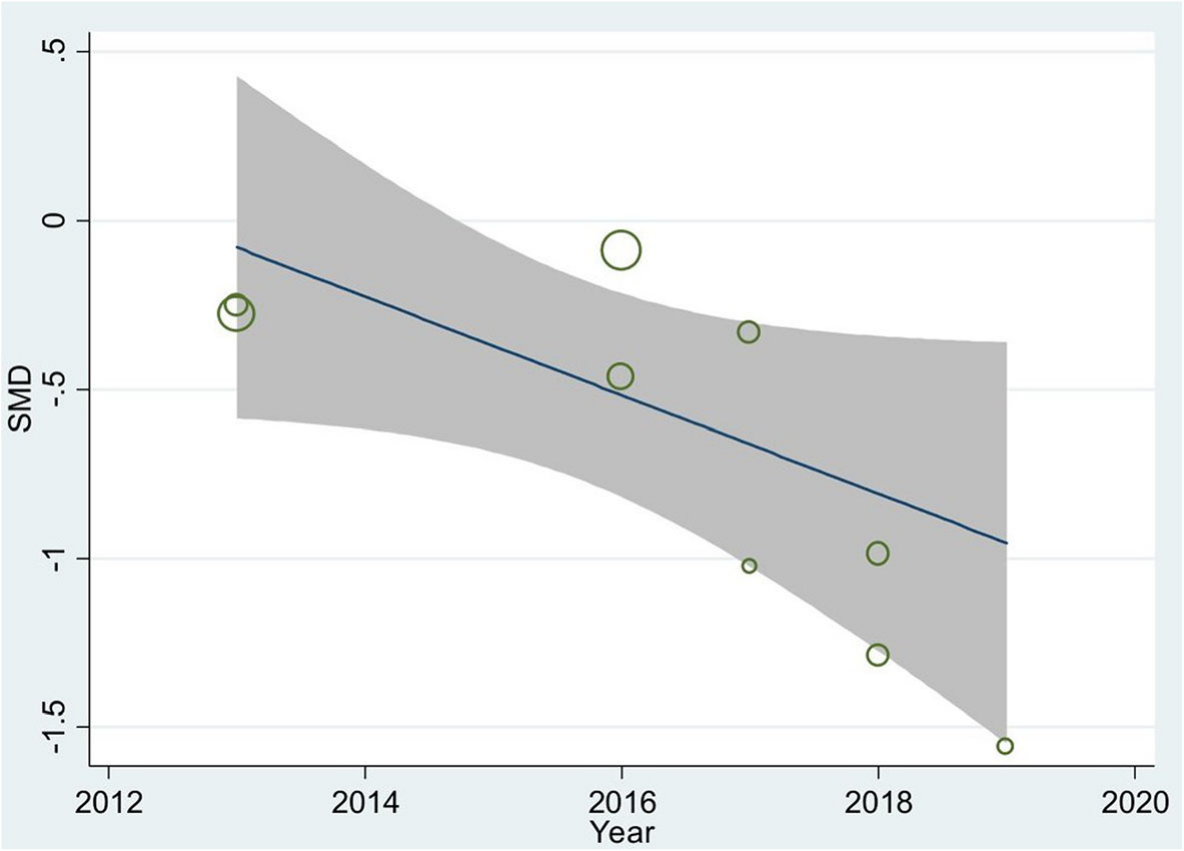
The meta-regression result of influencing publication year on the pooled SMD

Next, publication year and sample size were investigated as potential contributor’s variables using the meta-regression method; publication year (t = − 3.00, *p* = 0.02) was found to be significantly related to pooled SMD, but sample size was not (t = 2.31, *p* = 0.06). Moreover, it was found that the pooled SMD increased over time (Fig. 3).

**Fig. 4:**
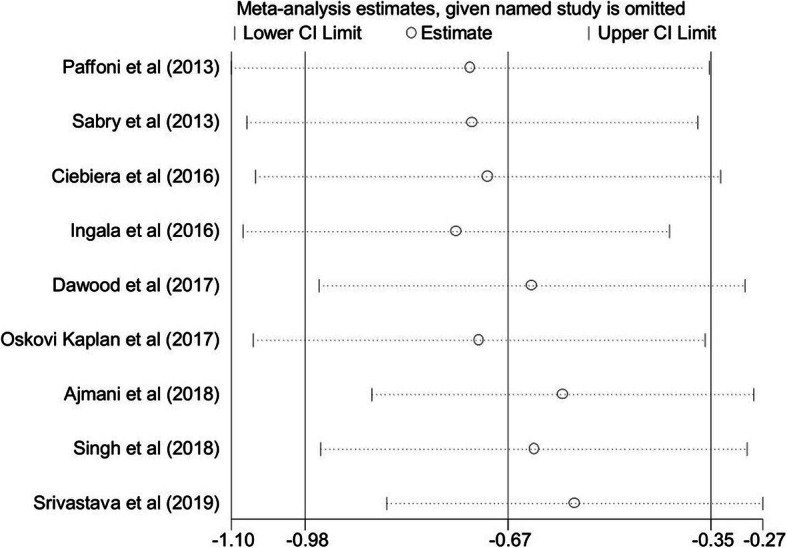
Sensitivity analysis result to assess each study on the pooled SMD

Sensitivity analysis indicated that the pooled SMD was not changed when each study was excluded (overall pooled SMD ranged between − 0.56 and − 0.75). The lower and higher pooled SMD for each study have been summarized in Fig. 4.

### Publication bias and quality assessment

The Begg’s (*p* = 0.03) and Egger’s (*p* = 0.01) statistics indicated that there was significant evidence for publication bias in the meta-analyses assessing the serum vitamin D levels in women with UL. However, we used the non-parametric to correct the findings (Duval and Tweedie methodology). The results showed no significant differences between before and after inclusion of the censored studies. The findings of quality assessment using the Newcastle-Ottawa scale are presented in Table 1.

## Discussion

Over the past few years, several studies have investigated the potential role of vitamin D or its active metabolites in the occurrence and development of UL. Both in vitro and in vivo studies have demonstrated that different doses of vitamin D can significantly shrink UL tumors [10, 28]. The reduction in UL size may be mediated by the suppression of cell growth and proliferation-related genes [10]. Additionally, it has been shown that paricalcitol, a vitamin D receptor activator, has the potential to reduce the proliferation of immortalized human UL cells [28].

It has been also suggested that vitamin D has the potential to suppress TGF-β-mediated activities which have been previously shown to contribute to regulating the proliferation of UL cells [9]. In line with basic research, a previous clinical study evaluating the effects of both circulating 25-hydroxyvitamin D concentration and sun exposure on UL occurrence and growth showed that both mentioned factors were inversely associated with UL growth [29].

This systematic review indicated that lower vitamin D levels are correlated with occurrence of UL; however, this finding needed to be confirmed in larger observational studies. Similarly, several studies have evaluated the relationship between vitamin D serum concentration and the odds of other types of gynecological benign and malignant tumors; however, their findings differed. A meta-analysis conducted by Yin et al. found a weak inverse association between serum vitamin D levels and the risk of ovarian cancer in women [30]. Another meta-analysis by Yan et al. indicated that there was no significant correlation between low vitamin D levels and the odds of various benign and malignant gynecological tumors [7]. After subgroup analysis, however, a significant association was found between low levels of vitamin D and benign gynecological tumors (OR = 0.97; *p*   =  0.047) in the latter meta-analysis [7].

Subgroup analysis in the current meta-analysis showed that heterogeneity decreased based on two factors: 1) different study area, and 2) publication year. The studies conducted in Africa represented a non-significant correlation between serum concentrations of vitamin D and the risk of UL, while this association remained statistically significant among Asian and European studies. This finding is in line with previous reports suggesting that a low vitamin D level was correlated with UL in white but not black populations, which may be attributable to differences in sun exposure, racial and individual factors [15, 31]. It is noteworthy to mention the two main factors influencing vitamin D production, i.e. skin pigmentation and geographical variation. Vitamin D deficiency is more common among Black Americans than other Americans living in northern America, mainly because pigmentation downregulates vitamin D production in the skin [32]. Moreover, the level of vitamin D among people living in Nordic latitudes has been shown to be lower than normal values because of diminished sunlight exposure [33].

The other factor leading to heterogeneity in this meta-analysis was publication year. Subgroup analysis showed that the inverse correlation between serum vitamin D level and odds of UL became more prominent among studies that had been published in recent years. This could be attributed to improved vitamin D deficiency detection methods. Subsequently, the detection rates of vitamin D deficiency among women with or without UL have risen [34]. The increasing trend of vitamin D deficiency in recent years could be due to several factors, such as lifestyle changes, being an indoor worker, urbanization, environmental pollution, and decreased outdoor activity [35–38]. Furthermore, low levels of physical activity correspond with an increasing risk of obesity, and obesity can directly increase the odds of UL through increased estrogen production by adipose tissue. It may also be indirectly involved in UL pathogenesis by decreasing the vitamin D concentration [39].

The current systematic review and meta-analysis and its publication bias assessment were limited by the relatively small number of eligible included studies. Therefore, the results should be interpreted with caution. However, results of the current meta-analysis could be used, for the first time, to shed light on the low vitamin D levels and increased risk of UL in women, while addressing limitations from previous observational studies. The authors strongly recommend that further studies with larger populations be conducted to further elucidate the current evidence.

## Conclusion

In summary, vitamin D deficiency is a possible risk factor for the occurrence of UL. Further studies with larger populations are definitely needed to evaluate vitamin D level in women with UL.

## Data Availability

The current review article was based on results of relevant published studies.

## Abbreviations

NOS: Newcastle-Ottawa scale
SMD: Standardized mean difference
TGF-β: Transforming growth factor beta
VDR: Vitamin D receptor
UL: Uterine leiomyoma
